# The Influence of Heat-Killed *Enterococcus faecium* BGPAS1-3 on the Tight Junction Protein Expression and Immune Function in Differentiated Caco-2 Cells Infected With *Listeria monocytogenes* ATCC 19111

**DOI:** 10.3389/fmicb.2019.00412

**Published:** 2019-03-05

**Authors:** Nikola Popović, Jelena Djokić, Emilija Brdarić, Miroslav Dinić, Amarela Terzić-Vidojević, Nataša Golić, Katarina Veljović

**Affiliations:** Laboratory for Molecular Microbiology, Institute of Molecular Genetics and Genetic Engineering, University of Belgrade, Belgrade, Serbia

**Keywords:** antilisterial effect, Caco-2 immune response, tight junction, *Enterococcus faecium* BGPAS1-3, heat-killed postbiotic

## Abstract

*Listeria monocytogenes*, the common foodborne pathogenic bacteria species, compromises the intestinal epithelial barrier, leading to development of the listeriosis, a severe disease especially among immunocompromised individuals. *L. monocytogenes* infection usually requires antibiotic treatment, however, excessive use of antibiotics promotes emergence of antibiotic resistance and the destruction of gut microbiota. Probiotics, including lactic acid bacteria (LAB), have been repeatedly proven as an alternative approach for the treatment of various infections. We have analyzed the potential of *Enterococcus faecium* BGPAS1-3, a dairy isolate exhibiting strong direct antilisterial effect, to modulate the response of differentiated Caco-2 intestinal epithelial cells to *L. monocytogenes* ATCC 19111 infection. We showed that the molecule with antilisterial effect is a bacterial cell-wall protein that is highly resistant to the high-temperature treatment. When we tested the antilisterial potential of heat-killed BGPAS1-3, we found that it could prevent tight junction disruption in differentiated Caco-2 monolayer infected with *L. monocytogenes* ATCC 19111, induce antilisterial host response mechanisms, and stimulate the production of protective TGF-β in intestinal epithelial cells. We also showed that the modulation of MyD88 dependent TLR2 and TLR4 pathways by BGPAS1-3 are involved in host response against *L. monocytogenes* ATCC 19111. Since heat-killed BGPAS1-3 possess strong antilisterial effects, such postbiotic could be used as a controllable and safe therapeutic.

## Introduction

Production and ripening of fermented products are accompanied with the high risk of contamination by foodborne spoilage and pathogenic bacteria, such as *Listeria monocytogenes*. *L. monocytogenes* can cause a serious disease called listeriosis that can lead to brain infection and death especially among pregnant women, the elderly and immunocompromised individuals (Ramaswamy et al., 2007). This infection begins with ingestion of contaminated food and one of the most critical steps in the course of the infection is the passage through the intestine barrier (Drevets and Bronze, 2008). A multi-protein complex between adjacent epithelial cells named tight junction forms selectively permeable intestinal epithelium barrier, which prevents different pathogens and toxins permeation (Berkes et al., 2003). *L. monocytogenes* expresses various factors, such as invasion protein internalin A (InlA), Listeria adhesion protein (LAP) (reviewed in Drolia et al., 2018), protein named InlC (Ireton et al., 2014), that compromise tight junction and other cell membrane protein complexes and form cell membrane pores and protrusions that allow bacteria invasion and multiplication in the host cells. In addition to barrier formation, intestinal epithelial cells are first that sense the presence of pathogens by recognition of different pathogen-associated molecular patterns (PAMPs) with different pathogen recognition receptors (PRRs), among which the Toll-like receptors (TLRs) are best characterized (Takeda and Akira, 2015). Ligation of these receptors initiates activation of epithelial cells to produce molecules with direct antimicrobial effects and the molecules that induce innate immune responses, which is an indispensable step in the sequence of events leading a successful clearance of the pathogen (Pamer, 2004). One of the most important pro-inflammatory cytokines produced by intestinal epithelial cells is IL-8 responsible for the chemotactic migration and activation of different immune cells at the site of infection (Onyiah and Colgan, 2016). On the other hand, intestinal epithelial cells produce transforming growth factor (TGF)-β, the molecule with prominent immunosuppressive effect (Rochman et al., 2009; Konkel and Chen, 2011) an important role in the maintenance of barrier integrity (Howe et al., 2005).

*L. monocytogenes* infection usually requires antibiotic treatment. The knowledge that excessive and improper use of antibiotics in human and veterinary medicine is closely related to the emergence of various side-effects such as antibiotic resistance and destruction of gut microbiota (Francino, 2015), forced search for an alternative approach for the treatment of different infections. Probiotics have been repeatedly proven to have the potential of improving host defense against pathogens (Fijan, 2014). As bacteria of the genus *Enterococcus* are mainly ancient and highly evolved members of the different animal intestinal tract as well as plants, soil, water, and various man-made products (Lebreton et al., 2014) it is reasonable to expect that they have developed different mechanisms that enable them struggling for the living space. In accordance with that, some enterococci strains produce one or more broad-spectrum antimicrobial compound(s) and may affect *L. monocytogenes* growth (Achemchem et al., 2006). In addition to antimicrobial effects, some enterococci could compete with pathogens for cell binding sites (Popovic et al., 2018). Also, enterococci are recognized by different PRRs, that could be used for immune responses modulation (Sparo et al., 2014; Carasi et al., 2017) and regulation of epithelial barrier function (Miyauchi et al., 2008). In addition, enterococci are used in the fermentation of dairy and meat products contributing to the ripening and aroma development of certain cheeses or fermented sausages, often those produced in Mediterranean countries (Franz et al., 2003; Morandi et al., 2013).

However, some enterococcal strains are associated with nosocomial infections, bacteremia, urinary tract infections, and endocarditis (Vu and Carvalho, 2011). Often, they can carry multiple antibiotic resistances (Veljovic et al., 2015; Anderson et al., 2017) and virulence factors such as cytolysin (Cyl, β-hemolysin), gelatinase (GelE), hyaluronidase (HylN) (Dworniczek et al., 2003).

As the consequence of such opposite features of different enterococcal strains, these bacteria belong to the most controversial lactic acid bacteria (LAB) (Giraffa and Sisto, 1997; Giraffa, 2003). Considering that, the enterococci represent the source of various biologically active molecules that could be very useful for the resolution of different diseases, but not forgetting their controversial status, the investigation of biological effects of non-live enterococcal preparations named postbiotics, could give a solution. In that aim, we previously isolated enterococci from fermented dairy products and tested their antimicrobial potential (Terzić-Vidojević et al., 2015). *E. faecium* BGPAS1-3 was isolated from traditional fresh soft cheese manufactured in households on a small scale in rural location surrounding Pale mountain city in Bosnia and Herzegovina and was selected for this study due to strong antilisterial effect. We showed that *E. faecium* BGPAS1-3 produces the cell wall protein with strong direct antilisterial effect. Interestingly, the similar direct antilisterial effect is retained after treatment of *E. faecium* BGPAS1-3 with high temperature (heat-killed BGPAS1-3). In addition to this direct antimicrobial effect, we showed that live and heat-killed BGPAS1-3 could prevent tight junction disruption, allows induction of *IL-8* mRNA and stimulate *TGF*-β mRNA expression in differentiated Caco-2 monolayer infected with *L. monocytogenes*.

## Materials and Methods

### Bacterial Strains, Medium, and Growth Conditions

*E. faecium* BGPAS1-3 from the laboratory collection of the Laboratory of Molecular Microbiology, Institute of Molecular Genetics and Genetic Engineering, University of Belgrade, Serbia was used in this study. *E. faecium* BGPAS1-3 was grown in M17 broth (Merck, GmbH, Darmstadt, Germany) supplemented with glucose (0.5% w/v) (GM17) at 37°C. *L. monocytogenes* ATCC 19111 was cultivated in Brain heart infusion (BHI) broth (Oxoid, Hampshire, United Kingdom) at 37°C. Corresponding agar plates were prepared by adding agar (1.7% w/v, Torlak, Belgrade, Serbia) into each broth.

### Preparation of *E. faecium* BGPAS1-3 Postbiotics

*E. faecium* BGPAS1-3 was cultured in GM17 broth (Merck) at 37°C under aerobic conditions. The supernatant (SN BGPAS1-3) of the overnight culture (ON) was collected after centrifugation of bacterial cells at 5000 rpm for 10 min, filtrated through 0.22 μm membrane filters and stored at -20°C for further analysis. In order to obtain live and heat-killed BGPAS1-3, bacterial pellet from ON cultures was washed two times with PBS and 10 times concentrated in PBS. Live BGPAS1-3 were used for the analysis of antilisterial activity and the treatment of Caco-2 cells. In order to obtain heat-killed BGPAS1-3 the part of live cells suspension was heated at temperatures of 60°C, 70, 80, 90, and 100°C for 30 min. The treatment of BGPAS1-3 with 100°C for 30 min was sufficient to kill all bacteria so we chose this treatment conditions to obtain heat-killed BGPAS1-3 postbiotic for all further experiments. Obtained heat-killed BGPAS1-3 lots were stored at -20°C for further analysis. In order to determine the number of live bacteria for heat-killed BGPAS1-3 preparation, before the high temperature treatment of bacterial PBS suspension, bacteria were plated on GM17 agar plates and enumerated after 24 h. For the analysis of antiliserial activity, all treatment (ON BGPAS1-3, live BGPAS1-3, SN BGPAS1-3, and heat-killed BGPAS1-3) were prepared from the same number of live bacteria. In all experiments with Caco-2 cells, the cells were treated with heat-killed BGPAS1-3 prepared from live bacteria at multiplicity of infection (MOI) of 10.

### PCR Detection of Virulence Determinants

The total DNA of BGPAS1-3 was used in PCR to detect the presence or absence of genes for virulence determinants and genes involved in biofilm formation. The primer sequences of the target genes, the expected amplicon sizes, and annealing temperatures are given in Table 1.

**Table 1:** List of the primers used in this study.

Genes	Primers	Product size	T°C	Reference
*hylN*	5′-ACAGAAGAGCTGCAGGAAATG-3′ 5′-GACTGACGTCCAAGTTTCCAA-3′	276 bp	56°C	Vankerckhoven et al., 2004
*agg*	5′-AAGAAAAAGAAGTAGACCAAC-3′ 5′-AAACGGCAAGACAAGTAAATA-3′	1553 bp	54°C	Eaton and Gasson, 2001
*cylA*	5′-TGGATGATAGTGATAGGAAGT-3′ 5′-TCTACAGTAAATCTTTCGTCA-3′	517 bp	58°C	Eaton and Gasson, 2001
*esp*	5′-TTGCTAATGCTAGTCCCAGACC-3′ 5′-GCGTCAACACTTGCATTGCCGAA-3′	933 bp	58°C	Eaton and Gasson, 2001
*efaA^fs^*	5′-GACAGACCCTCACGAATA-3′ 5′-ATGTCATCATGCTGTAGTA-3′	705 bp	56°C	Eaton and Gasson, 2001
*efaA^fm^*	5′-AACAGATCCGCATGAATA-3′ 5′-CATTTCATCATCTGATAGTA-3′	735 bp	56°C	Eaton and Gasson, 2001
*fsrA*	5′-ATGAGTGAACAAATGGCTATTTA-3′ 5′-CTAAGTAAGAAATAGTGCCTTGA-3	740 bp	43°C	Nakayama et al., 2002
*fsrB*	5′-GGGAGCTCTGGACAAAGTATTATCTAACCG-3′ 5′-TTGGTACCCACACCATCACTGACTTTTGC-3′	566 bp	43°C	Nakayama et al., 2002
*fsrC*	5′-ATGATTTTGTCGTTATTAGCTACT-3′ 5′-CATCGTTAACAACTTTTTTACTG-3′	1343 bp	43°C	Nakayama et al., 2002
*gelE*	5′-CGGAAGGCGTTACTGTTGAT-3′ 5′-GAGCCATGGTTTCTGGTTGT-3′	957 bp	46°C	Popovic et al., 2018
*sprE*	5′- TTGAGCTCCGTTCCTGCCGAAAGTCATTC-3′ 5′-TTGGTACCGATTGGGGAACCAGATTGACC-3′	591 bp	58°C	Nakayama et al., 2002


### Antimicrobial Activity Assay

Efficacy of antimicrobial compounds produced by BGPAS1-3 was tested by the deferred antagonism method using indicator strain *L. monocytogenes* ATCC 19111 (Lozo et al., 2004). The antilisterial effects of ON BGPAS1-3, live BGPAS1-3 cells washed with PBS three times as well as of prepared BGPAS1-3 postbiotics, supernatant (SN) BGPAS1-3 and heat-killed BGPAS1-3. To confirm the production of antimicrobial compounds of proteinaceous nature, crystals of pronase E (Sigma Chemie, GmbH, Germany) were placed close to the edge of the wells. After incubation for 24 h at 37°C a clear zone of inhibition around the well, but not near the pronase E, was taken as a positive signal for bacteriocin production.

### Cell Culture and Treatments

Differentiated human intestinal Caco-2 cells were used as an *in vitro* model of the intestinal epithelial barrier. Caco-2 cells were grown in DMEM supplemented with 10% fetal bovine serum (FBS), 100 μg/ml streptomycin, 100 U/ml penicillin, and 2 mM L-glutamine (Gibco, Thermo Fisher Scientific, Waltham, MA, United States). The cells were maintained in 75 cm^2^ flasks at 37°C in a humidified atmosphere containing 5% CO_2_. Cells were seeded in 24-well plate (approximately 2 × 10^5^ cells/well) and incubated at 37°C. After confluency, cells were left for 21 days to allow differentiation, as previously reported (Roselli et al., 2006).

To test the protective effect of live or heat-killed BGPAS1-3 against adhesion and invasion of *L. monocytogenes* ATCC 19111, both bacterial overnight cultures and previously prepared heat-killed cells were washed in PBS and resuspended in 0.5 ml DMEM in order to treat Caco-2 cells (1 × 10^6^) with BGPAS1-3 (live or heat-killed) and *L. monocytogenes* ATCC 19111 at a MOI of 10.

Three different assays were performed on differentiated Caco-2 cells: (1) for competitive assay, live or heat-killed BGPAS1-3 suspension and *L. monocytogenes* ATCC 19111 suspension was simultaneously added on differentiated Caco-2 cells, and incubated for 1 h at 37°C in a 5% CO_2_ incubator; (2) in the displacement assay, the *L. monocytogenes* ATCC 19111 was inoculated onto differentiated Caco-2 cells for 1 h, washed 3 times with PBS prior to 2 h inoculation of live or heat-killed BGPAS1-3; (3) in the exclusion assay, Caco-2 cells were treated with live or heat-killed BGPAS1-3 for 2 h, washed 3 times with PBS prior to 1 h inoculation with *L. monocytogenes* ATCC 19111.

In all assays, the cell cultures were washed three times with PBS to remove the non-adherent bacteria and then cells were detached with Trypsin-EDTA solution (Torlak, Belgrade, Serbia). The part of detached cells was used for adhesion assay of *L. monocytogenes* ATCC 19111 (Zivkovic et al., 2016) and calculated as the % of adhesion = [(CFU/ml a total number of counted bacteria – CFU/ml invaded bacteria)/ CFU/ml added bacteria] × 100. Part of the cells used for invasion analysis was incubated with gentamicin (500 μg/ml) for 30 min in order to kill extracellular adhered bacteria. Thereafter, cells were washed three times in PBS and lysed using 500 μl of cold 0.1% Triton X-100 and plated on BHI agar plates for enumeration of internalized bacteria. The % of invasion was calculated as (CFU/ml invaded bacteria/CFU/ml added bacteria) × 100. From the rest of the cells the total RNA and proteins were isolated, and stored at -80°C for quantitative real-time PCR analysis and at -20°C for Western blot analysis.

### Quantitative Real-Time PCR

The total RNA extraction from the Caco-2 cell was performed as previously described (Lukic et al., 2013) with minor modification. Briefly, the Caco-2 cells were lysed in denaturing solution. Thereafter, acid phenol (pH 4) extraction was performed followed by 2-propanol precipitation. Reverse transcription was done using 500 ng of isolated RNA as a template, according to the instructions of the enzyme manufacturer (Thermo Fisher Scientific). Random hexamers (Applied Biosystems, Foster City, CA, United States) and RiboLock RNase inhibitor (Thermo Fisher Scientific) were used in the reactions. Synthesized cDNA was amplified in 7500 real-time PCR system (Applied Biosystems) using KAPA SYBR Fast qPCR Kit (KAPA Biosystems, Wilmington, MA, United States) under the following conditions: 3 min at 95°C activation, 40 cycles of 15 s at 95°C and 60 s at 60°C. The results were normalized to endogenous control (β-*actin*) and expressed as relative target abundance using the 2^-ΔΔCt^ method. Primers used in the study are listed in Table 2.

**Table 2:** List of primers used for real time PCR analysis.

Genes	Primers	Reference
*TLR2*	5′-TGAGCTGCCCTTGCAGATAC-3′ 5′-TGCAAGCAGGATCCAAAGGA-3′	This study
*TLR4*	5′-GGATTTCACACCTCCACGCA-3′ 5′-GGTCAGAGCGTGATAGCGAG-3′	This study
*MyD88*	5′-CAGTTGCCGGATCTCCAAGT-3′ 5′-GTCTCCTCCACATCCTCCCT-3′	This study
*IL-8*	5′-ACACAGAGCTGCAGAAATCAGG-3′ 5′-GGCACAAACTTTCAGAGACAG-3′	Angrisano et al., 2010
*TGF*-β	5′-CCGGGTTATGCTGGTTGTACAG-3′ 5′-AAGGACCTCGGCTGGAAGTGG-3′	Dragicevic et al., 2017
*Claudin*	5′-TCACACATACCCTGTCAGGCT-3′ 5′-ATGGCCTCTCTTGGCCTCCAA-3′	Elamin et al., 2012
*Occludin*	5′-TCAGGGAATATCCACCTATCACTTCAG-3′ 5′-CATCAGCAGCAGCCATGTACTCTTCAC-3′	Elamin et al., 2012
β-*actin*	5′-TCAGTAACAGTCCGCCTAGAAGCA-3′ 5′-TTGCTGACAGGATGCAGAAGGAGA-3′	Li et al., 2015


### Western Blotting

For protein expression analysis the cells were lysed with RadioImmunoPrecipitation Assay buffer (50 mM Tris-HCl pH 7.4; 150 mM NaCl; 1% NP-40; 0.25% sodium deoxycholate) containing Protease inhibitor cocktail tablets (Roche, Basel, Switzerland) and 1 mM phenylmethylsulfonyl fluoride (Sigma-Aldrich, St. Louis, MO, United States), for 30 min on ice. The total protein concentrations were determined using the BCA protein assay kit (Thermo Fisher Scientific). Equal amounts of proteins (20 μg) were separated by 12.5% SDS-PAGE. Electrophoresed proteins were transferred from the gel to a 0.2 μm nitrocellulose membrane (GE Healthcare) using a Bio-Rad Mini *trans*-blot system (Bio-Rad, Hercules, CA, United States). Immunoblots were blocked in a 5% non-fat dry milk in TBS-Tween (50 mM Tris-HCl, pH 7.4; 150 mM NaCl, and 0.05% Tween-20) overnight at 4°C followed by 2 h incubation at room temperature with the primary antibodies anti-glyceraldehyde-3-phosphate dehydrogenase (GAPDH) (as a loading control; 1:1000; Beijing Dingguo Changsheng Biotechnology Co., Ltd.,) and anti-claudin (CLDN-4, 1:1000; Novus Biologicals, United States). The membranes were subsequently washed and incubated with appropriate HRP-conjugated secondary antibodies (goat anti-rabbit; 1:10,000; Thermo Fisher Scientific) for 1 h at room temperature. Proteins were detected by enhanced chemiluminescence (Immobilon Western, Merck Millipore).

### Statistical Analysis

All experiments were repeated at least three times independently, and each set of experiments was performed in triplicate. All data are presented as mean values ± standard deviation from different experiments. One-way ANOVA with the Tukey’s *post hoc* test was used to compare multiple groups. Values for *p* < 0.05 or less were considered to be statistically significant. Statistical analysis was carried out and graphs were prepared by using GraphPad Prism 5 software.

## Results and Discussion

### The Controversial Status of the *E. faecium* BGPAS1-3 Strain

Taking into account that mechanisms evolved by different strains of the highly diverse genus *Enterococcus* could be used against various medically important pathogens we previously isolated enterococci from fermented dairy products and tested their physiological properties that contribute to the technological process of fermented food production (Terzić-Vidojević et al., 2015).

In addition to human commensal bacteria, different species of genus *Enterococcus* are well characterized as human pathogens. In order to test the safety use of BGPAS1-3 as live probiotic, the presence of genes encoding different virulence factors in the genome of BGPAS1-3 was tested (Table 3). Enterococci colonize the gastrointestinal tract with adhesion factors binding to mucosal and other epithelial surfaces. In addition to the colonization of commensal bacteria, the adhesion to host tissues is considered a prerequisite for the establishment of infection (Koch et al., 2004). Enterococcal surface protein (encoded by *esp*), wall adhesins (encoded by *efaA^fs^*, and *efaA^fm^*), and aggregation substance (encoded by *agg*) are involved in the pathogenesis of *E. faecium* infections (Eaton and Gasson, 2001; Soares et al., 2014). The results of this study revealed the presence of the *esp, agg, efaA^fs^*, and *efaA^fm^* genes in the genome of BGPAS1-3. Although adhesion factors are involved in pathogenesis, our previous study suggested that the presence of the *agg* and *esp* genes positively correlate with characteristics related to probiotic potential (Popovic et al., 2018).

**Table 3:** Presence of virulence genes and the genes for biofilm formation.

Biofilm formation					Adhesins				Enzyme	
		
*fsrA*	*fsrB*	*fsrC*	*gelE*	*sprE*	*esp*	*agg*	*efaA^fs^*	*efaA^fm^*	*hylN*	*cylA*
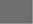	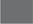		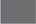	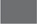	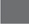	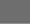	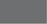	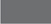		


*The shaded areas reflect the presence of the respective genes. fsrA, response regulator; fsrB, signaling peptide; fsrC, histidine kinase; gelE, gelatinase; sprE, serine protease; esp, enterococcal surface protein; agg, aggregation substance; efaA^*fs*^, cell wall adhesins (endocarditis specific antigen) expressed by E. faecalis; efaA^*fm*^, gene coding cell wall adhesins (endocarditis specific antigen) expressed by E. faecium; hylN, hyaluronidase; cylA, cytolysin activator.*

In addition, enterococci can form a biofilm that contributes to colonization and/or virulence. Biofilm is a structured and complex community of microorganisms adhering to the biotic or abiotic surface and is associated with bacterial mutual communication named quorum sensing system. We previously showed that BGPAS1-3 has no ability to form biofilm (Popovic et al., 2018) and now we tested the presence of genes involved in biofilm formation. Genes coding for proteins of the quorum sensing system are located in the *fsr* (fecal streptococci regulator) locus, consisting of the *fsrA, fsrB*, and *fsrC* genes. The presence of the complete *fsr* locus, including all three genes is necessary for biofilm formation. Importantly, the strain BGPAS1-3 carries the *fsrA* and *fsrB* genes, but not the *fsrC* gene, that is in accordance with the inability of this strain to form the biofilm. In general, the *fsr* locus is located next to the genes encoding the gelatinase (*gelE*) and the serine protease (*sprE*) and regulate their expression (Mohamed and Huang, 2007; Hashem et al., 2017). Bacteria that have serine protease and gelatinase activity provide nutrients by hydrolyzing different proteins like gelatin, casein, and hemoglobin (Koch et al., 2004; Banwo et al., 2013) which leads to degradation of host tissue, but also have the role in biofilm formation (Fisher and Phillips, 2009). Interestingly, although we previously showed that the strain BGPAS1-3 does not exhibit gelatinase and protease activity (Popovic et al., 2018), now we showed that this strain carries the *gelE* and *sprE* genes. This inconsistency between genotype and phenotype could be explained by the absence of the intact *fsrC* gene that has been characterized as a positive regulator of the *gelE* (Nakayama et al., 2002; Teixeira et al., 2012).

Further, we tested the presences of the *cylA* gene encoding CylA serine protease which is involved in processing and activation of cytolysin (also called haemolysin), a bacterial toxin with β-haemolytic properties in humans. Also the presence of *hylN* gene, encoding hyaluronidase, a degradative enzyme associated with tissue damage (Semedo et al., 2003) was analyzed. Importantly, the results revealed that none of these genes was detected in the genome of the strain BGPAS1-3 and this result is in accordance with the previously shown absence of hemolytic activity of the strain after 48 h incubation on blood agar plates (Popovic et al., 2018).

The concern that bacteria belonging to *Enterococcus* sp. could act as pathogens increased with the occurrence of high-level resistance to multiple antimicrobial drugs (Hollenbeck and Rice, 2012). Although the strain BGPAS1-3 was previously determined as antibiotic susceptible, according to the results obtained by disc diffusion method (Terzić-Vidojević et al., 2015), and by microdilution test, according to CLSI recommendations, BGPAS1-3 showed resistance to low levels of gentamicin, streptomycin, erythromycin, and ampicillin according to EFSA guidance (Popovic et al., 2018).

Taking into account that postbiotics could mimic the beneficial therapeutic effects of probiotics while avoiding the risk of administering live microorganisms expressing virulence factors, in this study the postbiotic properties of the heat-killed BGPAS1-3 were examined in comparison to live bacteria.

### The Strong Antilisterial Activity of the BGPAS1-3 Strain Is Retained After High-Temperature Treatment

**FIGURE 1:**
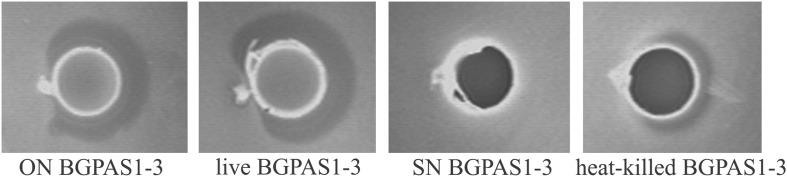
Direct antilisterial effect of overnight culture (ON) BGPAS1-3, live BGPAS1-3 cells, supernatant (SN) BGPAS1-3 and heat-killed BGPAS1-3 of *Enterococcus faecium* BGPAS1-3 was tested. The zone of *Listeria monocytogenes* ATCC 19111 growth inhibition around the well was taken as the positive signal of antimicrobial compounds production/activity. The confirmation of the proteinaceous nature of antimicrobial compounds is seen as a detectable growth at the edge of the inhibitory zone where the dot of pronase E crystals was placed.

Among the natural dairy isolates of the LAB, antibacterial mechanisms with a broad inhibitory spectrum were found in enterococci (Giraffa, 2003; Terzić-Vidojević et al., 2015). In accordance with that, BGPAS1-3 showed antimicrobial activity against *L. monocytogenes* ATCC 19111. Since BGPAS1-3 showed strong antilisterial effect (Figure 1), we further analyzed the effects of this enterococcal strain on Caco-2 cells infected with *L. monocytogenes* ATCC 19111. Considering the controversial status of enterococci, we tested the antilisterial effects of different fractions containing no BGPAS1-3 live cells such as filtrated SN (obtained by filtration with 0.22 μm membrane filters) and heat-killed BGPAS1-3 cells. Importantly, only heat-killed BGPAS1-3 retained part of the antilisterial activity as live strain pointing out that the cell wall components contribute to this effect. Importantly, we showed that the molecule with direct antilisterial effect in this fraction is protein partially resistant on high-temperature treatment. This results are in accordance with the previously published results of our group that *Lactobacillus salivarius* BGHO1 (Busarcevic and Dalgalarrondo, 2012) and *Lactococcus lactis* subsp. *lactis* bv. diacetylactis BGBU1-4 (Lozo et al., 2017) produce bacteriocin in cell-attached form that only could be separated from bacterial cells extract and not from supernatant. Proteinaceous nature of this cell-attached molecule with antilisterial effect has been confirmed by crystals of pronase E. These cell-attached bacteriocins as well as heat-killed BGPAS1-3 were shown to be thermostable and to retain antimicrobial activity over long periods of storage.

**FIGURE 2:**
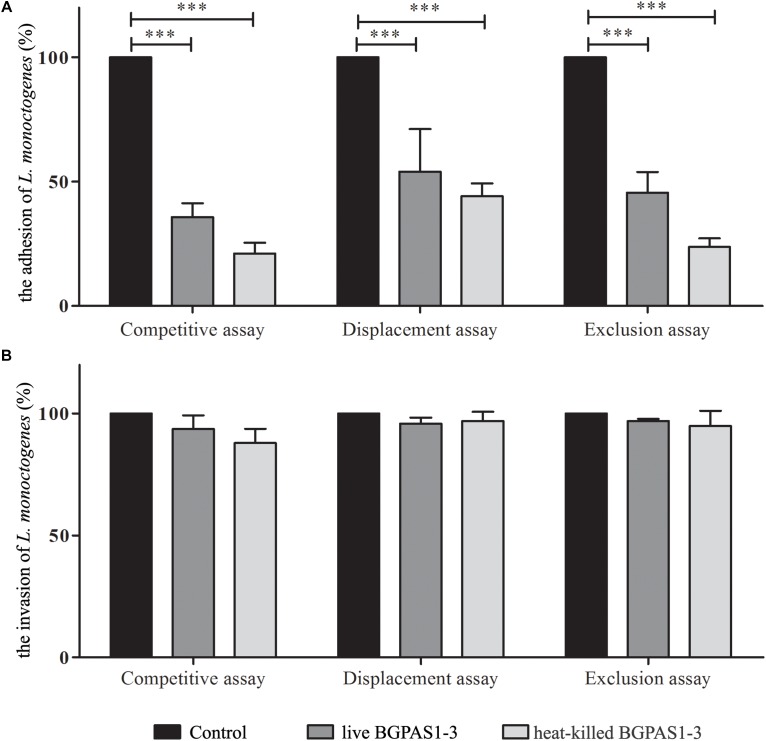
The effect of live and heat-killed *Enterococcus faecium* BGPAS1-3 on **(A)** adhesion and **(B)** invasion of *Listeria monocytogenes* ATCC 19111 on differentiated Caco-2 cells were analyzed in the competitive, the displacement and the exclusion assays. Three experiments were done. One-way ANOVA with the Tukey’s *post hoc* test was used to compare CFU of *L. monocytogenes* ATCC 19111 in cultures with and without BGPAS1-3. Statistical significance *p* < 0.001 was marked as ^∗∗∗^.

Thus, we compared the effects of this fraction with the effects of live BGPAS1-3 on antilisterial response of Caco-2 cells. In addition to the production of antimicrobial molecules, it was shown that adhesion of enterococci to intestinal epithelial cells enables their colonization on the intestinal mucosa and therefore limits the overgrowth of pathogens (Jin et al., 2000). Our previous study indicated that enterococci isolated from dairy products are able to adhere to the surface of intestinal epithelial cells (Popovic et al., 2018). In this study, we showed that about 70% of applied BGPAS1-3 adheres to Caco-2 cells after 2 h of co-cultivation. These results are in accordance with the results related to the other enterococcal surface proteins, such as Esp, Agg, AggE (Sartingen et al., 2000; Shankar et al., 2001; Veljović et al., 2017), as well as di-glucosyl-di-acyl-glycerol/lipoteichoic acid (LTA) (Sava et al., 2009), which allow binding to the intestinal epithelium. Considering that, we assessed the effectiveness of live or heat-killed BGPAS1-3 on adhesion and invasion ability of *L. monocytogenes* ATCC 19111. We found that live or heat-killed BGPAS1-3 strongly inhibited adhesion of *L. monocytogenes* ATCC 19111 to differentiated Caco-2 cells during competition (35.8% ± 5.5 and 21.1% ± 4.4), exclusion (45.5% ± 8.4 and 23.7 ± 3.5), and displacement (46.4 ± 4.9 and 44.1% ± 5.2) (Figure 2A). Although live and heat-killed BGPAS1-3 have shown to reduce *L. monocytogenes* ATCC 19111 adhesion, none of the fractions has the ability to reduce the invasion of *L. monocytogenes* ATCC 19111 in Caco-2 cells (Figure 2B). Well-known role of heparin and heparan sulfate expressed on epithelial host cells in the binding of enterococcal di-glucosyl-di-acyl-glycerol/LTA (Sava et al., 2009) and listerial protein, ActA (Suárez et al., 2001) indicates these molecules as the place of BGPAS1-3 and *L. monocytogenes* ATCC 19111 competition. These data point to the possibility that in addition to the strong direct bactericidal antilisterial effect of BGPAS1-3, a decrease in *L. monocytogenes* ATCC 19111 adhesion on Caco-2 cells could be due to the competition of bacteria and bacterial molecules/components for the same adhesion ligands. Considering these promising results on the potential protective effect of BGPAS1-3 as live probiotic as well as the heat-killed postbiotic in the case of listerial intestinal infection, it would be very interesting to test if these probiotic/postbiotic BGPAS1-3 preparation have the same effect in an animal model of listerial infection.

### The Effects of BGPAS1-3 on Antilisterial Response of Caco-2 Cells

**FIGURE 3:**
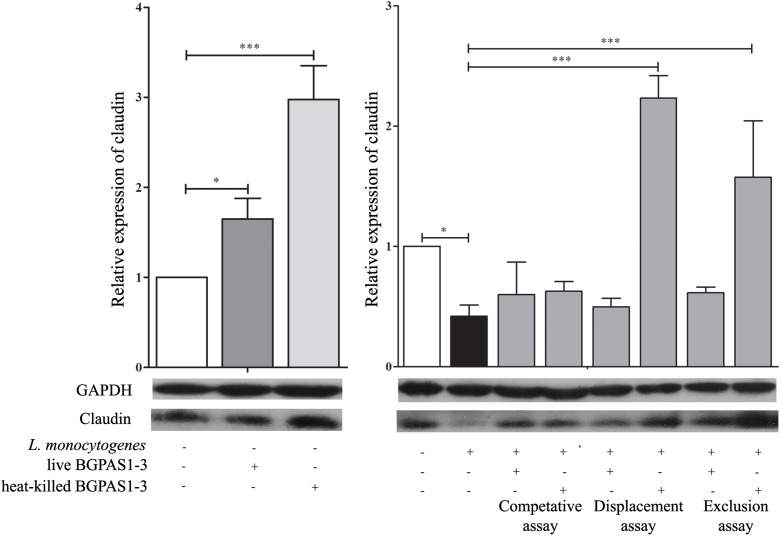
The effect of *Listeria monocytogenes* ATCC 19111, live and heat-killed *Enterococcus faecium* BGPAS1-3 on *claudin* expression by differentiated Caco-2 cells was analyzed on mRNA (qPCR, graphs) and protein (the representative images of Western blot, GAPDH was used as housekeeping protein) level in the competitive, the displacement and in the exclusion assays. Three experiments were done. One-way ANOVA with the Tukey’s *post hoc* test was used to compare the expression of mRNA for claudin (relative to β-*actin* as housekeeping gene) in untreated cultures with BGPAS1-3 treated and *L. monocytogenes* ATCC 19111 treated cultures, as well as in *L. monocytogenes* ATCC 19111 treated cultures with BGPAS1-3/*L. monocytogenes* ATCC 19111 treated cultures. Statistical significance *p* < 0.001, *p* < 0.05 was marked as ^∗∗∗^, ^∗^, respectively.

Bacterial pathogens, including *L. monocytogenes*, can disrupt the tight junction transmembrane structures and cause epithelial barrier dysfunction (Drolia et al., 2018). The different tight junction proteins form multifunctional transmembrane complex involved in intestinal homeostasis (Zihni et al., 2016). Claudins are the major determinant of the barrier function of tight junctions and it was shown that decrease in expression is correlated with different human diseases (Hadj-Rabia et al., 2004; Usami et al., 2006; Lee, 2015). Accordingly, we showed that *L. monocytogenes* ATCC 19111 infection of Caco-2 cells strongly decreases claudin expression at mRNA and protein level (Figure 3). On the other hand, treatment of uninfected differentiated Caco-2 cells with live or heat-killed BGPAS1-3 strongly stimulated the expression of claudin. In accordance to such protective role of BGPAS1-3, there was no significant decrease in claudin expression in *L. monocytogenes* ATCC 19111-infected Caco-2 cells treated with various fractions of BGPAS1-3. Interestingly, only heat-killed BGPAS1-3 stimulated the expression of this molecule when Caco-2 cells were treated before or after *L. monocytogenes* ATCC 19111 infection. In addition to claudin we showed that heat-killed BGPAS1-3 has stimulatory effect on expression of mRNA for occludin, another important tight junction protein in Caco-2 cells (Supplementary Figure S1). At the other hand, the infection with *L. monocytogenes* ATCC 19111 showed no effect on expression of occludin mRNA in Caco-2 cells so we did not analyzed the effect of BGPAS1-3 in the infected Caco-2 cells. This effect on tight junction proteins point to the potential protective role of BGPAS1-3 probiotic/postbiotic treatment in the case of intestinal infection. In order to confirm this assumption, it is necessary to examine the impact of these treatments on intestinal barrier function by using the equipment for transepithelial electrical resistance measurement in our further research.

**FIGURE 4:**
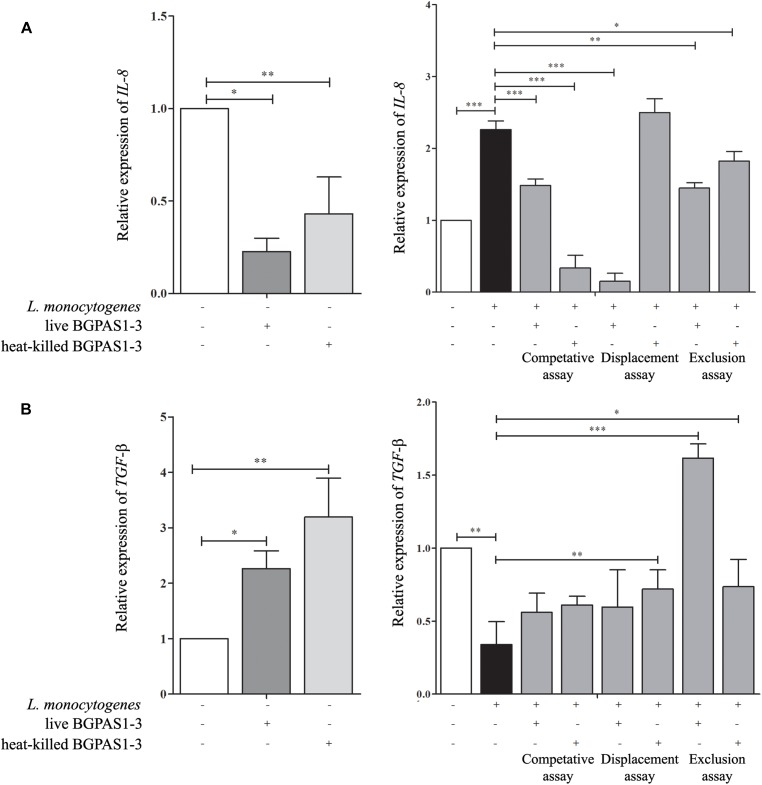
The effect of *Listeria monocytogenes* ATCC 19111, live and heat-killed *Enterococcus faecium* BGPAS1-3 on **(A)** *IL-8* and **(B)** *TGF*-β mRNA expression by differentiated Caco-2 cells was analyzed in the competitive, the displacement and the exclusion assays. Three experiments were done. One-way ANOVA with the Tukey’s *post hoc* test was used to compare the expression of mRNA for *IL-8* and *TGF*-β (relative to β-*actin* as housekeeping gene) in untreated cultures with BGPAS1-3 treated and *L. monocytogenes* ATCC 19111 treated cultures, as well as in *L. monocytogenes* ATCC 19111 treated cultures with BGPAS1-3/*L. monocytogenes* ATCC 19111 treated cultures. Statistical significance *p* < 0.001, *p* < 0.005, *p* < 0.05 was marked as ^∗∗∗^, ^∗∗^, ^∗^, respectively.

Although the primary function of intestinal epithelial cells is mechanical protection, these cells have an important role in the recognition of pathogen which leads to their activation and consequently to induction of an acute immune response. IL-8 secreted by several cell types, including epithelial cells, has an important role in the attraction and activation of leukocytes (Onyiah and Colgan, 2016). Moreover, it has been shown that IL-8 initiates an acute inflammatory response in listeriosis (Opitz et al., 2006). In accordance with that, infection of differentiated Caco-2 cells with invasive *L. monocytogenes* ATCC 19111 in our setting increased *IL-8* mRNA expression (Figure 4A). On the other hand, after the exposure of differentiated uninfected Caco-2 cells to live or heat-killed BGPAS1-3, level of *IL-8* mRNA was decreased (Figure 4A). Furthermore, live or heat-killed BGPAS1-3 treatments of Caco-2 cells before *L. monocytogenes* ATCC 19111 infection decreased significantly the level of *IL-8* mRNA expression in comparison to treatment with *L. monocytogenes* ATCC19111 alone. The same results were obtained when the Caco-2 cells were treated simultaneously with live or heat-killed BGPAS1-3 and *L. monocytogenes* ATCC 19111. Interestingly, when *L. monocytogenes* ATCC 19111 infected Caco-2 cells were treated thereafter with heat-killed BGPAS1-3 the level of *IL-8* mRNA induced by *L. monocytogenes* ATCC 19111 remained unchanged (Figure 4A). Taking into account indispensable role of IL-8 in the effective antilisterial host response, as well as the importance of intestinal barrier integrity, this enterococcal postbiotic exhibits potential to prevent bacterial translocation and spreading of *L. monocytogenes* infection and could be considered as safe enough treatment for listeriosis.

In addition to the important role of IL-8 in antilisterial host defense, the unrestrained inflammation could induce disruption of epithelial barrier (Naydenov et al., 2013). Therefore, the simultaneous activation of immunosuppressive mechanisms that control ongoing inflammation is of crucial importance for the harmless and successful pathogen elimination. In that mean, TGF-β can protect the epithelial barrier function by preventing inflammation-mediated epithelial damage and up-regulating of the tight junction protein claudin-1 (Rachakonda et al., 2016). Also, it was shown that TGF-β pretreatment could protect T84 monolayers against barrier dysfunction induced by enterohemorrhagic *Escherichia coli* O157:H7 (Howe et al., 2005). Infection of Caco-2 cells with *L. monocytogenes* ATCC 19111 decreased *TGF*-β mRNA expression in our experiments (Figure 4B). Oppositely, exposure of uninfected differentiated Caco-2 cells to live or heat-killed BGPAS1-3 elevated level of *TGF*-β mRNA expression. In accordance to that, the treatment of Caco-2 cells with heat-killed BGPAS1-3 after *L. monocytogenes* ATCC 19111 infection and with live or heat-killed BGPAS1-3 treatment before *L. monocytogenes* ATCC 19111 infection increased significantly the level of *TGF*-β mRNA expression in comparison to treatment with *L. monocytogenes* ATCC 19111 alone. Considering the important role of TGF-β in the suppression of unrestrained inflammation and in the maintenance of intestinal epithelial barrier, this effect of BGPAS1-3 postbiotic could contribute to the protection of epithelial barrier function during listeriosis.

### TLR2, TLR4, and MyD88 Are Involved in Modulation of Caco-2 Functions by BGPAS1-3

**FIGURE 5:**
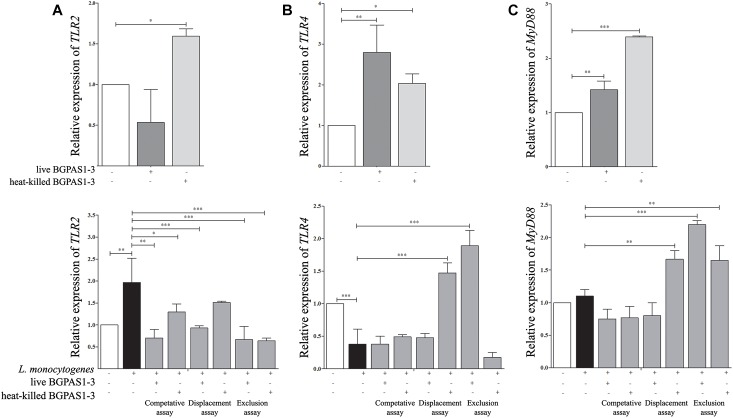
The effect of *Listeria monocytogenes* ATCC 19111, live and heat-killed *Enterococcus faecium* BGPAS1-3 on **(A)** *TLR2*, **(B)** *TLR4*, and **(C)** *MyD88* mRNA expression by differentiated Caco-2 cells was analyzed in: the competitive, the displacement and the exclusion assays. Three experiments were done. One-way ANOVA with the Tukey’s *post hoc* test was used to compare the expression of mRNA for *TLR2, TLR4*, and *MyD88* (relative to β-*actin* as housekeeping gene) in untreated cultures with BGPAS1-3 treated and *L. monocytogenes* ATCC 19111 treated cultures, as well as in *L. monocytogenes* ATCC 19111 treated cultures with BGPAS1-3/*L. monocytogenes* ATCC 19111 treated cultures. Statistical significance *p* < 0.001, *p* < 0.005, *p* < 0.05 was marked as ^∗∗∗^, ^∗∗^, ^∗^ respectively.

The fine balance of homeostasis and defense responses in intestinal tissue is regulated with simultaneous recognition of different microbial stimuli by different TLR (McClure and Massari, 2014). TLRs are dominantly expressed by immune cells, but also these receptors are expressed on epithelial cells and induce cell activation in a MyD88-dependent or independent manner. Recognition of *L. monocytogenes* peptidoglycans by TLR2 in MyD88-dependent fashion was shown to be required for induction of innate immune response against this pathogen in mice model of infection (Torres et al., 2004). In addition to this, it is hypothesized that TLR4 might have a role in recognizing surface LTA of live *L. monocytogenes* in the MyD88-independent way (Edelson and Unanue, 2002). In accordance to such primary role of TLRs, it was shown that manipulation of TLR expression by probiotic strains could modulate the immune response in different diseases (Castillo et al., 2011; Villena and Kitazawa, 2014; Lépine and de Vos, 2018). As we expected, in our study infection of differentiated epithelial cells with *L. monocytogenes* ATCC 19111 stimulated *TLR2* mRNA expression (Figure 5A). In addition to *L. monocytogenes*, the immune response to *E. faecium* was shown to be dependent on TLR2 activation (Leendertse et al., 2008). Interestingly, only heat-killed BGPAS1-3 stimulated expression of this receptor in Caco-2 cells. As a result, the level of *TLR2* mRNA expression was decreased in all treatments, except the unchanged level of this mRNA in the Caco-2 cells treated with heat-killed BGPAS1-3 after *L. monocytogenes* ATCC 19111 infection. On the other hand, infection of Caco-2 cells with *L. monocytogenes* ATCC 19111 suppressed the *TLR4* mRNA expression. Interestingly, although the treatment of Caco-2 with live or heat-killed BGPAS1-3 alone stimulated the level of *TLR4* mRNA expression, the expression of this receptor increased only in Caco-2 cells treated with live BGPAS1-3 after *L. monocytogenes* ATCC 19111 infection or with heat-killed BGPAS1-3 before *L. monocytogenes* ATCC 19111 infection (Figure 5B). In addition, the pattern of changes in *MyD88* mRNA expression showed a strong correlation with the changes of expression of mRNA for TLRs in cultures (Figure 5C). Thus, stimulation of *TLR2* and suppression of *TLR4* mRNA expression in Caco-2 by *L. monocytogenes* ATCC 19111 infection resulted in an unchanged level of *MyD88* mRNA in comparison to control Caco-2. The stimulation of *MyD88* mRNA expression in Caco-2 treated with BGPAS1-3 was consistent with the stimulation of TLR2 and TLR4 in these cultures. In accordance, expression of *Myd88* mRNA increased significantly with stimulation of both TLRs mRNA expression in Caco-2 cells treated with heat-killed BGPAS1-3 after *L. monocytogenes* ATCC 19111 infection. In addition, stimulation of the only TLR4 in Caco-2 cells treated with live BGPAS1-3 before *L. monocytogenes* ATCC 19111 infection are correlated with the increase in *MyD88* mRNA expression. More pronounced effect of heat-killed BGPAS1-3 in comparison to live bacteria is probably the result of the temperature-induced liberation of different active molecules originating inside live bacteria or sheltered in 3D structures of complex molecules on the bacterial surface. Another research group (Sultana et al., 2013) previously described similar mechanisms.

## Conclusion

Considering that heat-killed BGPAS1-3 possess strong direct antilisterial effect, simultaneously allowing the induction of immune mechanisms important for antilisterial host response and stimulates the production of protective TGF-β in intestinal epithelial cells, we assume that the application of this enterococcal postbiotic in the case of listerial infection could significantly contribute to *L. monocytogenes* clearance with minimum harmful effects on host organism. In addition to soluble bacterial products, heat-killed bacteria could be considered as postbiotic that could be used as a more controllable and safer therapeutics.

## Data Availability

All datasets generated for this study are included in the manuscript and/or the Supplementary Files.

## Author Contributions

KV, NG, and JD conceived and designed the study. NP and JD performed the main work. AT-V participated in the research-virulence traits, antibiotic susceptibility, and antimicrobial activity. NP, KV, and JD participated in the research Caco2 cell culture experiments. MD and EB performed and analyzed western blot and qPCR. All authors finally approved the version to be published.

## Conflict of Interest Statement

The authors declare that the research was conducted in the absence of any commercial or financial relationships that could be construed as a potential conflict of interest.
