# Supplementary material for: The Influence of Heat-Killed Enterococcus faecium BGPAS1-3 on the Tight Junction Protein Expression and Immune Function in Differentiated Caco-2 Cells Infected With Listeria monocytogenes ATCC 19111
Source: Front Microbiol. 2019 Mar 5;10:412. doi: 10.3389/fmicb.2019.00412 (PMC6411766; doi:10.3389/fmicb.2019.00412)
Supplement: Supplementary file 1 [file Data_Sheet_1.docx]

**
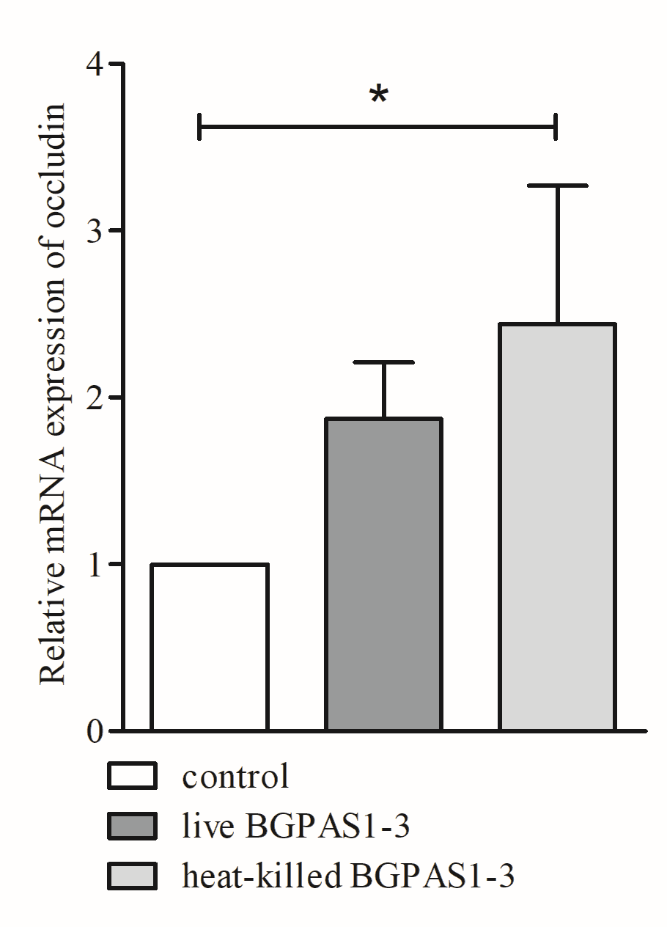
Figure S1.** The effect of live and heat-killed *Enterococcus faecium* BGPAS1-3 on occludin mRNA expression by differentiated Caco-2 cells. Three experiments were done. One-way ANOVA with the Tukey’s post hoc test was used to compare the expression of mRNA for occludin (relative to β-actin as housekeeping gene) in untreated cultures with BGPAS1-3 treated. Statistical significance p<0.05 was marked as *.
